# P-1292. Clinical and Genomic Features of Colistin Resistance Among Carbapenem-resistant Acinetobacter baumannii Isolated From Global Clinical Samples

**DOI:** 10.1093/ofid/ofaf695.1480

**Published:** 2026-01-11

**Authors:** Madison Stellfox, Lauren Komarow, Robin Patel, David van Duin, Daria Van Tyne, Yohei Doi

**Affiliations:** University of Pittsburgh, Pittsburgh, PA; George Washington University, Rockville, Maryland; Mayo Clinic, Rochester, MN; University of North Carolina at Chapel Hill, Chapel Hill, NC; University of Pittsburgh School of Medicine, Pittsburgh, Pennsylvania; University of Pittsburgh, Pittsburgh, PA

## Abstract

**Background:**

Carbapenem-resistant *Acinetobacter baumannii* (CRAb) is a multi-drug resistant, opportunistic pathogen. Colistin is one of a few remaining treatment options, but resistance exists globally.

**Methods:**

Between 2017 and 2019, 551 CRAb isolates were collected through the international Study Network of *Acinetobacter* as a Carbapenem-Resistant Pathogen (SNAP). Demographics, clinical characteristics, mortality, and a desirability of outcome ranking (DOOR) measure combining lack of clinical response, unsuccessful discharge, renal failure, and *C. difficile* infection, were recorded for each patient. A central laboratory measured colistin minimum inhibitory concentrations (MICs), and each isolate was classified as colistin-intermediate (MIC ≤2µg/mL) or resistant (MIC ≥4µg/mL). All isolates were sequenced on the Illumina platform, and the genomes were used to construct a phylogenetic tree and assess for variants within known colistin resistance-related genes.

**Results:**

18% of the 551 isolates were colistin-resistant. Compared to patients with colistin-intermediate isolates, patients with resistant strains were more likely to have been admitted from a healthcare facility, but otherwise had similar demographics and Charlson comorbidity indices. Focusing on patients with bloodstream or respiratory infections (n=217) uncovered increased 30-day mortality in patients with monomicrobial, colistin-resistant infections (11/19, p=0.008) when compared to monomicrobial-intermediate (41/102), polymicrobial-resistant (4/20) and polymicrobial-intermediate infections (18/76). Comparative genomics indicated that the majority of isolates belonged to clonal complex 2 (CC2), and that colistin resistance concentrated in certain genetic lineages. Variant analysis uncovered nonsynonymous mutations near and within the histidine kinase domain of *pmrB*, part of the PmrAB two-component system that regulates lipopolysaccharide modifications which can alter colistin susceptibility.

**Conclusion:**

In this global cohort, patients with monomicrobial, colistin-resistant CRAb infections had increased mortality. New treatment options are needed for multidrug-resistant CRAb, and this study provides further evidence that *pmrB* may serve as a worthwhile drug development target.

**Disclosures:**

Madison Stellfox, MD, PhD, Cumberland Pharmaceuticals: Grant/Research Support Robin Patel, MD, a patent on Bordetella pertussis/parapertussis PCR issued, a patent on a device/method for sonication, a patent on PET imaging of bacterial infection: a patent on Bordetella pertussis/parapertussis PCR issued, a patent on a device/method for sonication, a patent on PET imaging of bacterial infection|MicuRx Pharmaceuticals and bioMérieux: Grant/Research Support|PhAST, Day Zero Diagnostics, DEEPULL DIAGNOSTICS, S.L., Nostics, HealthTrackRx, bioMérieux and CARB-X: Advisor/Consultant|Up-to-Date and the Infectious Diseases Board Review Course: Honoraria David van Duin, MD, PhD, British Society for Antimicrobial Chemotherapy: Editor stipend|Merck: Advisor/Consultant|Merck: Grant/Research Support|Pfizer: Advisor/Consultant|Roche: Advisor/Consultant|Shionogi: Advisor/Consultant Yohei Doi, MD, PHD, GSK: Advisor/Consultant|Meiji Seika Pharma: Advisor/Consultant|Shionogi: Advisor/Consultant|Shionogi: Honoraria

